# Metacognitive Functioning and its Relationship with Clinical Insight and Functional Outcomes in Schizophrenia: The Role of Illness Duration

**DOI:** 10.1192/j.eurpsy.2025.324

**Published:** 2025-08-26

**Authors:** C. Brasso, A. Carluccio, G. Colli, C. Montemagni, R. Sgro, P. Rocca

**Affiliations:** 1 Neuroscience, University of Turin, Turin, Italy

## Abstract

**Introduction:**

Understanding the complex interplay between cognitive and functional domains in schizophrenia is crucial for improving patient functional outcomes.

Metacognition has emerged as a critical factor in the functioning and rehabilitation of individuals with schizophrenia. Additionally, the role of disorganization and clinical insight in this complex disorder has been extensively studied due to their role in shaping in the trajectory and real-life functioning outcomes of the disorder.

**Objectives:**

This study aimed to assess the differences and relationships between disorganization, clinical insight, metacognition, and various functional domains in patients with short (< 5 years) and long (≥ 5 years) illness duration.

**Methods:**

A network representation of the relationships between variables and the calculation of centrality and clustering indices were performed within each group. The two groups were compared with a network comparison test.

**Results:**

Eighty-nine patients with early and one hundred and six with late phase SZ were included. We found no significant differences in the overall network structure between the two groups despite patients with longer illness duration showing significant worsening across all examined domains. Interestingly, the relationships between the network variables remained stable regardless of disease duration.

One notable difference emerged in the correlation between interpersonal functioning and unawareness of symptoms, negatively correlated only in the group with longer illness duration. These results suggest that the link between social functioning and clinical insight may become more pronounced as the disorder progresses. Decentration was more central in the group with short disease duration, whose patients do not yet show marked impairment of metacognition, particularly in the ‘understanding other’s mind’ domain. Regarding clustering indices in the group with short illness duration, the misattribution of symptoms to the disorder is more clustering. On the other hand, metacognitive mastery, was highly clustering in both the short and long illness duration.

**Image 1:**

**Figure:**
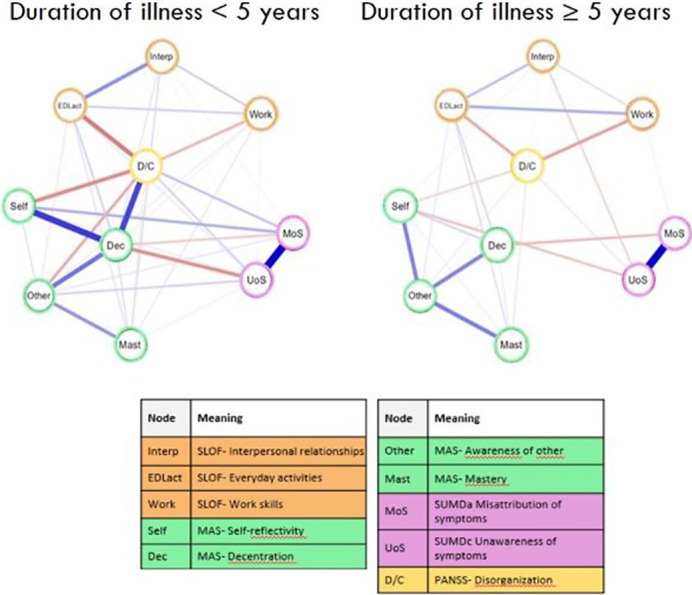

**Conclusions:**

The stability of the relationships between metacognitive variables, clinical insight, and functional outcomes, regardless of disease duration, suggests that interventions targeting metacognition may be beneficial for improving functioning in patients with schizophrenia. Additionally, the emerging negative correlation between interpersonal functioning and unawareness of symptoms in patients with longer illness duration highlights the importance of addressing both social skills and clinical insight as the disease progresses. These findings underscore the potential utility of integrated treatment approaches that address cognitive, social, and functional domains to optimize functional outcomes for individuals with schizophrenia across the course of the illness.

**Disclosure of Interest:**

None Declared

